# A Recombinant Snake Cathelicidin Derivative Peptide: Antibiofilm Properties and Expression in *Escherichia coli*

**DOI:** 10.3390/biom8040118

**Published:** 2018-10-22

**Authors:** Mercedeh Tajbakhsh, Maziar Mohammad Akhavan, Fatemeh Fallah, Abdollah Karimi

**Affiliations:** 1 Pediatric Infections Research Center (PIRC), Research Institute for Children Health, Shahid Beheshti University of Medical Sciences, Tehran 1546815514, Iran; mercedeh.tajbakhsh@gmail.com or m_tajbakhsh@sbmu.ac.ir (M.T.); fafallah@sbmu.ac.ir (F.F.); 2 Skin Research Center, Shahid Beheshti University of Medical Sciences, Tehran 1989934148, Iran; m_akhavan@sbmu.ac.ir

**Keywords:** antimicrobial peptide, anti-biofilm, cathelicidin, expression, fusion protein

## Abstract

The emergence of antimicrobial resistance among pathogenic microorganisms has been led to an urgent need for antibiotic alternatives. Antimicrobial peptides (AMPs) have been introduced as promising therapeutic agents because of their remarkable potentials. A new modified cathelicidin-BF peptide (Cath-A) with 34 amino acid sequences, represents the potential antimicrobial effects against methicillin-resistant *Staphylococcus aureus* (MRSA) with slight hemolytic and cytotoxic activities on eukaryotic cells. In this study, the effects of Cath-A on *Acinetobacter baumannii*, and *Pseudomonas aeruginosa* isolated from medical instruments were studied. Cath-A inhibited the growth of bacterial cells in the range of 8–16 μg/mL and 16-≥256 μg/mL for *A. baumannii* and *P. aeruginosa*, respectively. The peptide significantly removed the established biofilms. To display a representative approach for the cost-effective constructions of peptides, the recombinant Cath-A was cloned in the expression vector pET-32a(+) and transformed to *Escherichia coli BL21*. The peptide was expressed with a thioredoxin (Trx) sequence in optimum conditions. The recombinant peptide was purified with a Ni^2+^ affinity chromatography and the mature peptide was released after removing the Trx fusion protein with enterokinase. The final concentration of the partially purified peptide was 17.6 mg/L of a bacterial culture which exhibited antimicrobial activities. The current expression and purification method displayed a fast and effective system to finally produce active Cath-A for further in-vitro study usage.

## 1. Introduction

Microbial drug resistance is considered a global public health problem. The spreading of multidrug-resistant (MDR) bacteria threatens the healthcare system. The increasing cost of treatment, prolonged hospitalization, and failure to prevent serious infections are affected by the overuse of antibiotics [1,2]. Because of the lack of effective antimicrobial agents, the medical procedures for immune-compromised patients such as those needing organ transplantation, cancer chemotherapy, diabetes management, and major surgery become seriously high risk [3]. For these reasons, in 2014, the World Health Organization emphasized the urgent need to develop new alternative antimicrobial agents for a post-antibiotic era [4]. Among the MDR pathogenic bacteria, *Acinetobacter baumannii* and *Pseudomonas aeruginosa* are among the common causes of morbidity and mortality in hospitals [5,6,7].

These non-fermenting Gram-negative pathogens are quickly resistant to almost all antibiotic classes such as β-lactam, aminoglycosides, and quinolones agents because of their improved intrinsic resistant mechanisms [8,9,10]. Recent publications have reported the prevalence of extensively drug resistance *Acinetobacter* and *Pseudomonas* spp. in nosocomial infections all around the world [11,12,13,14,15]. There are many reports of nosocomial infections associated with the MDR *Acinetobacter* and *Pseudomonas* spp. from Iran [16,17,18,19]. Regarding the current world-wide reports, the World Health Organization (WHO) categorized these mentioned bacteria in a critical group of MDR bacteria which need new antibiotic agents urgently [20,21]. The ability of *Acinetobacter* and *Pseudomonas* spp. to attach and form biofilms on both biotic and abiotic surfaces is a critical role to their pathogenesis in hospitalized patients. Their ability to form MDR microbial biofilms causes a varied range of infections such as skin, wound, and urinary tract infections, to septicemia in immune-suppressed or immune-compromised patients [22,23,24]. Because of the physiological properties of the microbial biofilms, bacterial masses are more resistant to various conventional antibiotic agents. Their increased resistance to antimicrobial agents (up to 1000-fold compared to planktonic cells), making the treatment of biofilm-associated infection extremely challenging. Recently, biofilm-associated infections have emerged as a major problem in clinical settings. It seems that the current clinical antibiotics do not have the potential to combat and remove microbial biofilms [25,26]. Therefore, the discovery of potentially powerful new compounds with novel mechanisms of action to eradicate biofilm-forming cells needs to be developed.

One of the alternative agents to conventional antibiotics is antimicrobial peptides (AMPs), which have been under focus regarding their potential activities against microorganisms. The AMPs are defined as small molecules (10–50 amino acids) with a positive net charge (+2–+9) that exhibit amphipathic properties. They have critical roles in modulating the innate immune system of the host’s defenses [27,28,29].

AMPs are generally bactericidal and exhibit broad-spectrum antimicrobial effects against Gram-positive and Gram-negative bacteria via the electrostatic interaction between the negatively charged microbial cell surfaces (lipopolysaccharide and lipoteichoic acid of Gram-negative and Gram-positive bacteria, respectively) and the positively charged peptides [30,31]. They inhibit bacterial growth via membrane disruption or pore formation or efflux the entire cell’s content. Some AMPs have intracellular targets. They pass through the cell membrane and bind to their targets. During this process, critical biological processes including cell wall formation or DNA, RNA, and protein synthesis, are inhibited and lead to cell death [32,33]. In addition, it has been shown that many AMPs can prevent the biofilm formation or remove the attached bacterial biofilms due to different specific mechanisms, which include the inhibition of bacterial cell attachment to the surfaces, inducing the motility gene expression, the down-regulation of extracellular matrix synthesis, inhibition quorum sensing, and rapid bacterial killing ability [34,35,36].

Nevertheless, there are some limitations for the clinical applications of AMPs, such as their potential toxicity to human cells, the susceptibility to proteases, and the high cost of industrial peptide (>20 residues in length) synthesis [32,37]. In order to obtain a large quantity of AMPs for further analysis, the recombinant production strategies are utilized. The bacterial expression system is a great candidate for this purpose, owing to its rapid growth, cost effectiveness, and the different accessibilities of commercial vectors. The cloning of AMP genes in a suitable vector towards fusion proteins has been developed so as to cover the toxicity of the expressed cationic peptides for the host microorganism and to protect the AMPs from the proteases [38,39]. 

Cathelicidins are a major group of cationic antimicrobial peptides and have been detected in the immune system of several vertebrates. The cathelicidin’s structure consists of two different regions: a cathelin-like domain at the N-terminus that displayed a high similarity, at the intra-species and a heterologous domain at the C-terminus that represented the antimicrobial activities [40]. 

Recently, a new type of cathelicidin was determined in the venom glands of the *Bungarus fasciatus* snake (Cath-BF), which exhibited potential antimicrobial activity against the MDR pathogenic bacteria, with minimal hemolytic and cytotoxic effects on human cells [41,42]. It is well defined that by increasing the net positive charge of cationic peptides, the interaction of the AMPs with the negatively bacterial cell membrane is developed [43,44]. As reported previously, through the substitution of the positively charged amino acid (lysine) in the peptide’s sequence, the peptide (Cath-A: KRFKKFFRKLKKSVKKRKKEFKKKPRVIKVSIPF) displayed a high efficacy against the bacteria, with decreased hemolytic and cytotoxic exhibitions on the eukaryotic cells [45]. 

The possible application of developed AMPs as anti-biofilm agents on biomaterial surfaces is useful in the hospital setting. They can exert for combating MDR and/or biofilm forming bacterial infections in the healthcare system [46]. 

In the present study, Cath-A was used for anti-biofilm tests. In order to establish cost-effective production with the potential activity of the peptide, the recombinant Cath-A (rec-Cath-A) gene sequence was expressed by the *E. coli* utilization system. The developed expression systems in *E. coli* have provided an opportunity to produce large quantities of various AMPs [47,48,49,50]. However, codon optimization problems, a lethal toxicity of the expressed peptides to the *E. coli* host, instability of AMPs against bacterial proteases, the correct fold of expressed products, are challenges to achieve a biologically active form of AMPs. For this reason, several fusion partners have been introduced to facilitate the expression and purification of AMPs [38,39]. 

According to the nature of the expressed peptide, the plasmid of pET-32a with a T_7_ promoter and TrxA as a fusion partner was applied in order to reduce the toxicity of the AMPs to the host cell and to express the foreign protein in a soluble form [51]. This procedure was followed by an enzymatic cleavage (enterokinase) which resulted in the production of an intact Cath-A sequence. The antimicrobial activity of the recombinant peptide was examined against two strains of *A. baumannii* and *P. aeruginosa.*

## 2. Materials and Methods

### 2.1. Materials

The peptide with a purity ≥90% was synthesized by GL Biochem Ltd. (Shanghai, China). The antibiotic powders (ampicillin, piperacillin, levofloxcacin, imipenem, ceftazidime, tetracycline), enterokinase, isopropyl β-d-1-thiogalactopyranoside (IPTG) and chemical reagents were purchased from Sigma Aldrich Co. (St. Louis, MO, USA). The antibiotic disks prepared from MAST House (Merseyside, UK). The colistin disk was prepared from Rosco (Taastrup, Denmark). The Luria–Bertani (LB) broth and Mueller–Hinton (MH) broth (Pronadisa, Madrid, Spain), and the Tryptic Soy Broth (TSB), LB and MH agar (Merck KGaA, Darmstadt, Germany) were prepared. The polymerase chain reaction (PCR) reagents and plasmid extraction kit were purchased from Bioneer (Daejeon, South Korea). All of the chemicals that were used were of the analytical grade. The expression vector pET-32a (+) was purchased from Novagen (Billerica, MA, USA). *E. coli* strain DH5α was used for sub-cloning and plasmid amplification. The *E. coli Bl21 (DE3)* was a host for the expression transformed plasmids. The clinical bacterial isolates were prepared by the Milad Hospital, Tehran, Iran. The ATCC standard controls were kindly donated by the referral laboratory of Iran’s Ministry of Health and Medical Education. The HisTrap FF 5 mL column (GE Healthcare Europe GmbH, Freiburg, Germany) was used as an affinity chromatography for the purification of the histidine-tagged recombinant protein.

### 2.2. Clinical Bacterial Strains

The MDR *P. aeruginosa* and *A. baumannii* isolates were collected for the current study during three months. All of the bacteria were isolated from the clinical instruments (catheters, ventilators, etc.) of the patients who were submitted to the intensive care unit (ICU) of the Milad Hospital, Tehran, Iran. The bacterial strains were confirmed by standard microbiological tests. The antibiotic susceptibility tests against the conventional antibiotics were performed by the disk diffusion method based on the Clinical and Laboratory Standards Institute (CLSI) [52]. The strains were assessed against the following antibiotic disks: piperacillin (100 μg), ceftazidime (30 μg), cefepime (30 μg), imipenem (10 μg), gentamicin (10 μg), amikacin (30 μg), tetracycline (30 μg), and ciprofloxacin (5 μg). All of the *P. aeruginosa* isolates were tested against colistin (10 μg). The minimum inhibitory concentration (MIC) of the isolates was also tested using the broth microdilution method against ampicillin, tetracycline, levofloxacin, ceftazidime, cefepime, piperacillin, and imipenem based on the CLSI recommendation [52]. The *E. coli ATCC 25922* and *P. aeruginosa* ATCC 27853 were used as controls for the antibiotic tests.

### 2.3. Antimicrobial Activity of the Peptide

The antimicrobial activity of the synthetic peptide was tested against the clinical and standard isolates by the modified broth microdilution method as described previously [53]. Briefly, the bacterial strains were cultured in a TSB medium at 37 °C overnight. The fresh bacteria were diluted in MH broth. Serial doubling dilutions of the peptide, with a range of 1–512 μg/mL, were prepared in 0.01% acetic acid and 0.2% bovine serum albumin (BSA). A total of 50 μL of the peptide dilution was added to a 96-well plate, which was followed by 50 μL of bacterial suspensions added to each well in order to give a final 2 × 10^5^ colony-forming unit (CFU)/mL inoculum. The plates were incubated for 24 h at 37 °C. The MH broth with untreated bacterial suspension was used as the positive control. The negative controls were in un-inoculated media with normal saline. The MIC of the peptide was defined as the minimum concentration which inhibited the bacterial growth visually. All of the experiments were performed in triplicates.

### 2.4. Investigation of Antimicrobial Effects on an Established Biofilm

Two clinical strains *P. aeruginosa* no. 1 and *A. boumannii* no. 1 (according to Table 1) were selected to assess the biofilm inhibitory effect of the peptide in a 96-well flat bottom tissue culture plate (TCP), with a few modifications [54,55]. Briefly, the bacteria were cultured overnight in an MH broth and then diluted in the same medium, which was supplemented with 0.2% glucose. Then, 100 μL of 2 × 10^5^ CFU/mL of bacterial suspensions were added to the individual wells of polystyrene TCP and were incubated at 37 °C for 24 h in order to allow for the biofilm formation. The media containing normal saline were defined as the negative control. During the following day, the plates were washed three times with phosphate-buffered saline (PBS) 1× to remove the non-attached cells. The established biofilms were treated with 10 μL of 10× concentrations of antimicrobial agents (Cath-A and levofloxacin) to reach a final concentration of 4–256 μg/mL and 90 μL of fresh MH broth. The bacterial suspension without any treatment agents was performed as the positive control. The plates were incubated for 24 h at 37 °C. Following the incubation time, the plates were washed with PBS so as to remove the planktonic cells and left to dry at room temperature. For the fixation of the biofilms, 100 µL of 100% methanol was added for 10 min, then, by removing the methanol, the wells were stained with 100 μL of 0.1% crystal violet (cv) for 15 min. The TCPs were washed with sterile distilled water and were left to dry. Finally, 100 μL of glacial acetic acid (30% *v*/*v*) was added to dissolve the cv. The absorbance of the stained biofilm was measured at an optical density (OD) of 595 nm by a microplate reader (BioTek Instruments, PowerWave XS, Winooski, VT USA). The experiments were conducted in triplicates.

### 2.5. Recombinant Vector Construct

**Figure 1:**
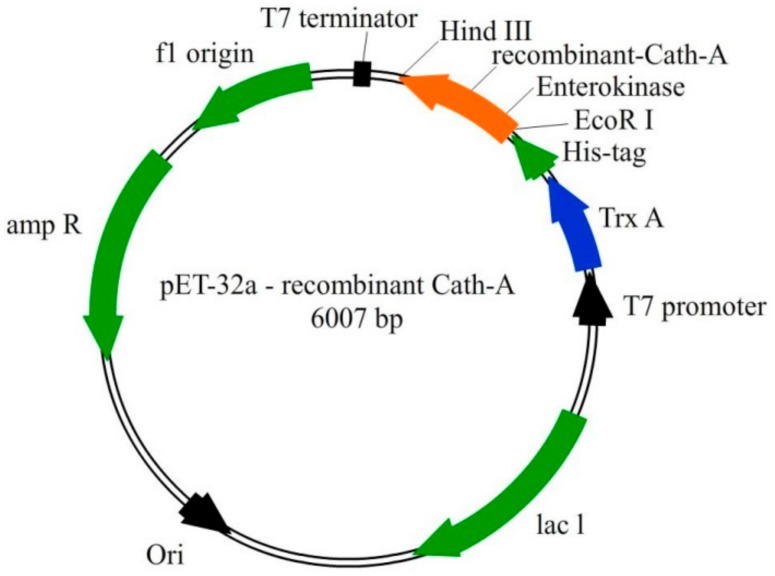
The schematic representation of the pET-32a vector construction. The recombinant Cath-A was expressed with the thioredoxin (TrxA) fusion partner, 6 His-tag, and enterokinase sequences. The 34 amino acids sequence of Cath-A with an additional enterokinase site (at the N terminus) was inserted into the *EcoRI* and *HindIII* restriction sites.

The entire gene sequence, including the rec- Cath-A with the additional enterokinase cleavage site was designed, as seen in Figure 1. The whole construct was synthesized by Bioneer Company (Daejeon, South Korea). According to the *E. coli* expression system, the codon optimization was done and the gene was inserted into pET-32a as an expression vector. The recombinant plasmid (pET-32(a)—rec Cath-A) and non-recombinant (original vector of pET-32(a)) were transformed into competent *E. coli* DH5α cells. The positive transformed cells were confirmed by the colony PCR. The PCR tests were performed using T_7_ universal primers. The PCR products were purified with a PCR product purification kit and were sequenced to exhibit the fidelity of the transformed plasmids by an ABI 3130 Genetic Analyzer (Applied Biosystems, Foster City, CA, USA). The plasmids of the positive competent cells were extracted using a Bioneer plasmid mini extraction kit and were transformed into competent *E. coli Bl21 (DE3)* cells.

### 2.6. Expression of the Fusion Protein

To optimize the highest protein expression, different media cultures, IPTG concentration, incubating times, and temperatures after the IPTG induction, were tested. 

A fresh colony of transformed *E. coli BL21 (DE3)* with expression vector was inoculated in 50 mL of the 2XYT medium containing 100 mg/L of ampicillin and were cultured in a shaking incubator at 37 °C for 12 h. Then, 10 mL of the culture was transferred to 500 mL of a fresh Terrific broth (TB) medium containing 100 mg/L ampicillin and 1% glucose. The bacteria were cultured at 37 °C with 200 rpm shaking until the optical density (OD 600) reached 0.6. The protein expression was induced by IPTG to the final concentration of 1 mM. The transformed cells were cultured for a further 6 h of cultivation at 25 °C. Subsequently, the cells were harvested by centrifugation at 5000 rpm, at a temperature of 4 °C for 10 min. The bacterial pellets were re-suspended in the binding buffer (20 mM NaH_2_PO_4_, 500 mM NaCl, 20 mM imidazole, pH 7.5) and were lysed on ice by sonication for 10 cycles (20 s working and 40 s rest). The supernatant of the lysate was collected by centrifugation at 14,000 rpm for 20 min at 4 °C. The extracted proteins were detected by a 15% sodium dodecyl sulfate-polyacrylamide gel electrophoresis (SDS–PAGE) and were stained with coomassie brilliant blue R-250.

### 2.7. Recombinant Proteins Isolation and Purification

The proteins were applied to His Trap FF columns in order to detect the His-tag proteins. The column was washed with the binding buffer several times. The extracted proteins were loaded on a column and were eluted by a gradient of the imidazole concentration (0–100%) from an elution buffer (20 mM NaH_2_PO_4_, 500 mM NaCl, 500 mM imidazole; pH 7.5). The peak fractions were collected and concentrated overnight at 4 °C by polyethylene glycol (PEG) with a 20,000 molecular weight. The concentrated proteins were analyzed on SDS-PAGE.

The fraction, which contained the recombinant peptide (rec-Cath-A) were dialyzed against a 500-mL volume of the enterokinase buffer (10 mM Tris HCI, pH 8.0, with 10 mM CaCl_2_) overnight at 4 °C.

### 2.8. Proteolytic Cleavage of Fusion Protein

The rec- Cath-A fusion protein was incubated with enterokinase at a ratio of 5 U:1 µg of the fusion protein in 10 mM of Tris HCI, pH 8.0, and 10 mM CaCl_2_. The reaction mixture was incubated at 37 °C overnight. After the cleavage process, the reaction was subjected to amicon ultra-centrifugal filters (Merk, Darmstad, Germany) with 30 kDa as well as a 3 kDa molecular weight cut-off in order to remove the enterokinase and non-specific proteins. The peptide solution was dialyzed (14 kDa cut-off) in 5 mL of PBS the 1X buffer in order to achieve a released Cath-A peptide and was analyzed by SDS-PAGE.

### 2.9. Analysis of Protein Concentration

The standard Bradford protein assay was displayed for the quantitative determination of the purified protein concentration [56].

### 2.10. Antimicrobial Activity Assay of Expressed Peptide

The antimicrobial activity of the purified peptide was determined using the agar disk diffusion method, based on the standard assay which was recommended by the CLSI against standards and clinical MDR bacteria [52]. 

Briefly, the 0.5 Mcfarland of the fresh colonies of bacteria were sub-cultured on MH agar and blank disks with different concentrations of synthetic peptide were placed on the plate. Approximately 20 μL of the dialyzed peptide was inoculated on the blank disk. The plates were incubated at 37 °C overnight. The inhibition zones were measured and compared to the synthetic peptide. All of the experiments were performed in triplicates. 

### 2.11. Statistical Analysis

The experiments were performed in triplicates. Data were analyzed by one-way analysis of variance (ANOVA), and standard deviations of the mean (mean ± SD) were presented for each test. *p* values < 0.05 and 0.01 were indicated as significant. 

## 3. Results

### 3.1. Antimicrobial Activities of Antibiotics and Peptide

Clinical isolates exhibited resistance to all antibiotic disks except *P. aeruginosa* isolates that were susceptible to colistin in the disk diffusion method (Figure S1). The MIC value of commercial antibiotics and Cath-A synthetic peptide against bacteria are presented in Table 1. As the results showed, the bacteria were resistant to all antibiotic agents. In comparison to conventional antibiotics, Cath-A exhibited a potential antimicrobial activity against bacterial isolates. The MIC values of the serial dilution of Cath-A were 16->256 μg/mL and 8–32 μg/mL for *P. aeruginosa* and *A. baumannii*, respectively. The peptide could inhibit *A. baumannii* isolates greater than commercial antibiotics with an average inhibitory concentration of approximately 12.8 μg/mL. This value is noteworthy compared to the MIC of other tested antibiotic agents. In the case of *P. aeruginosa*, some were not inhibited by Cath-A and were resistant to the peptide (MIC ≥ 256 µg/mL). The average inhibitory concentration effect of the peptide (134 μg/mL) was greater than levofloxacin (65 µg/mL) and imipenem (82 µg/mL) for clinical *P. aeruginosa*.

### 3.2. Disruption of the Established Biofilms

**Figure 2:**
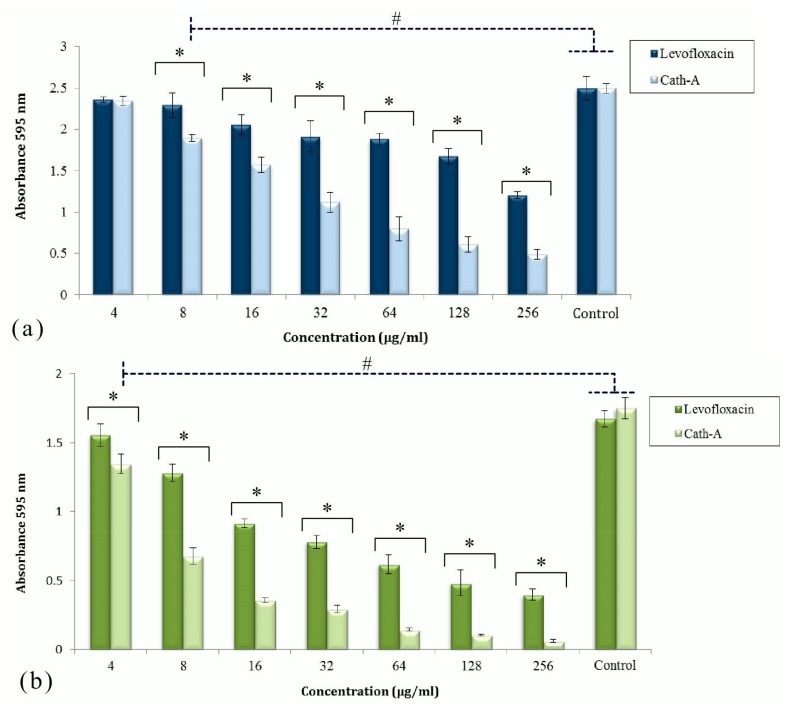
The activities of levofloxacin and Cath-A against the established biofilms of *P. aeruginisa* (**a**) and *A*. *baumannii* (**b**) at different concentrations (4–256 µg/mL). The antibiofilm effect of the peptide and levofloxacin was assessed by cv staining after 24 h of incubation. Biofilm formation was determined by measuring the absorbance at 595 nm using a microplate reader. Controls were untreated inoculated bacteria in Mueller Hinton (MH) broth. Data are shown as the mean ± SD of three independent tests. Significant different values were (# *p* < 0.05) vs controls and (* *p* < 0.01) with antimicrobial agents.

The effects of Cath-A and levofloxacin against biofilm structures formed by the MDR isolates in the TCP assay are displayed in Figure 2. Two strains, *P. aeruginosa* 1 and *A. baumanii* 1 without mucoidal surfaces and a level of resistance to levofloxacin were collected for the biofilm formation in polystyrene plates. After the biofilm formation and antimicrobial treatment, the plates were stained by 0.1% cv and OD 595 nm was read by a micro-plate reader. The amounts of OD revealed the adhered bacterial biomass. According to these findings, among the untreated bacteria, the growth of *P. aeruginosa* was much higher than *A. baumannii*.

The activities of Cath-A and levofloxacin to remove the planktonic cells were significant compared to the control cells (*p* < 0.05). Cath-A could remove a considerable amount of the attached biomass of *P. aeruginosa* in comparison to levofloxacin at concentrations ≥128 µg/mL (*p* < 0.01). With respect to this analysis, Cath-A could reduce the *A. baumannii* biofilm at lower concentrations. The antibiofilm effects of the peptide to decrease the *A. baumannii* biomass were considerable at ≥8 µg/mL concentrations. The Cath-A peptide could eradicate almost all of the *A. baumannii* attached cells in the biofilm at concentrations ≥64 µg/mL, in contrast to levofloxacin.

### 3.3. Expression of Recombinant Peptide in E. coli BL21

**Figure 3:**
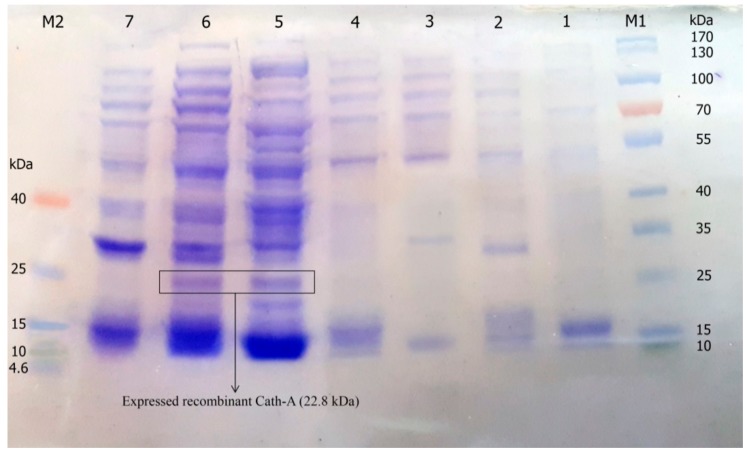
The expression of recombinant Cath-A in *E. coli BL21* and 1 mM inducted isopropyl β-d-1-thiogalactopyranoside (IPTG) in the Luria–Bertani (LB) broth medium. M1: pre-stained protein ladder 10–180 kDa; lane 1: total protein before IPTG induction; lane 2–7: total protein after 3–8 h of IPTG induction at 25 °C; M2: pre-stained low range protein ladder (1.7–40 kDa).

**Figure 4:**
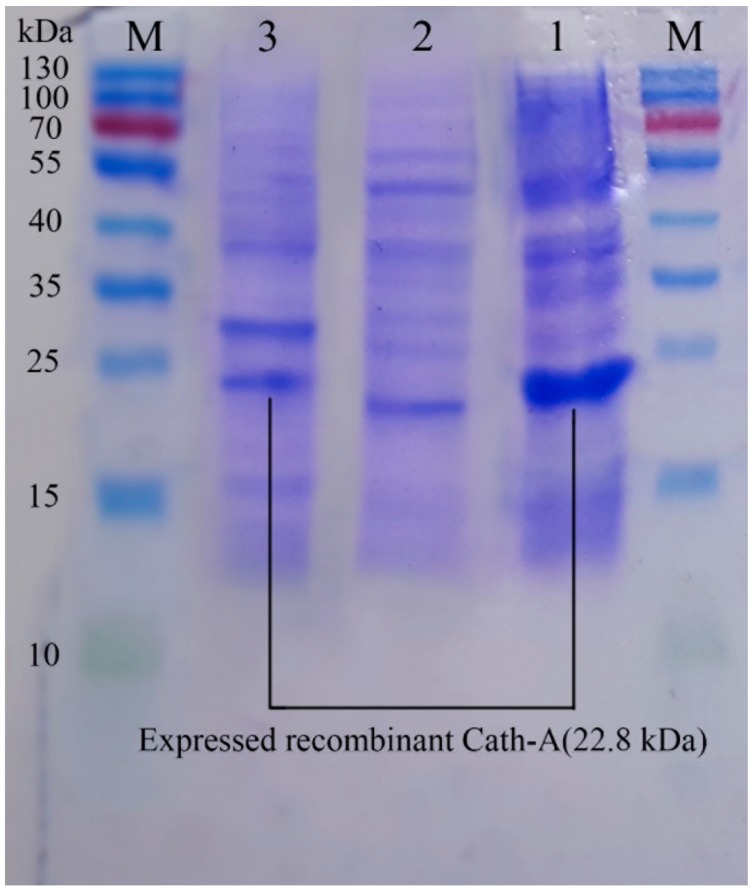
The sodium dodecyl sulfate polyacrylamide gel electrophoresis (SDS–PAGE) analysis of the expressed recombinant Cath-A in *E. coli BL21* and 1 mM inducted IPTG in the Terrific broth (TE) medium. M: pre-stained protein ladder 10–180 kDa; lane 1: the expressed total protein of the recombinant vector including rec-Cath-A after 6 h of IPTG induction at 25 °C; lane 2: the expressed total protein of the original vector (pET-32a); lane 3: the expressed total protein of the recombinant vector including rec-Cath-A after 7 h of IPTG induction at 25 °C.

The construct of the recombinant plasmid (pET 32a +rec-Cath-A) with (approximately) a 22.8 kDa molecular weight, including the non-toxic fusion protein (Trx), His-tag sequence for affinity chromatography, enterokinase cleavage site the for peptide release, and the Cath-A sequence (Figure 1) was confirmed with the DNA sequencing of the PCR product. The recombinant plasmid was transformed into *E. coli* BL21 (DE3) for the expression of the fusion protein by the T_7_ promoter. Time and medium compositions were major factors which were evaluated to achieve large scales of production yields. The soluble products were expressed in the TE medium after 6 h of 1 mM IPTG induction at 25 °C. Increasing the time factor to more than 6 h after IPTG induction, led to the decrease of the peptide production. The expression of the constructed vector including the recombinant peptide (~22.8 kDa) in various expression conditions was analyzed by SDS-PAGE (Figure 3 and Figure 4). 

### 3.4. Purification of Recombinant–Cath-A

**Figure 5:**
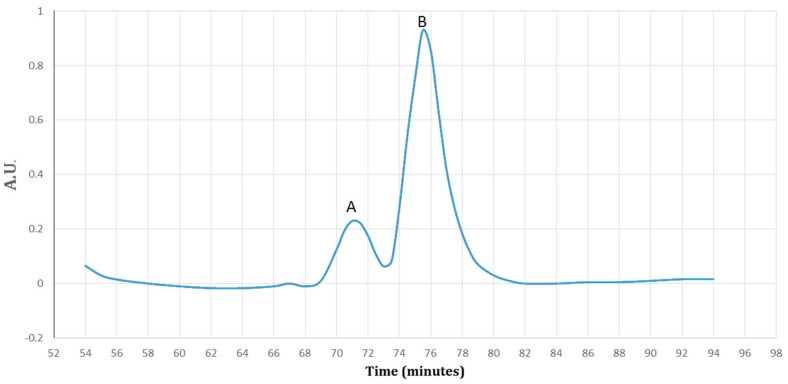
A diagram of the purified protein fractions by a gradient of the imidazole (0–100%). Fraction A detected at 17% (85 mM) and fraction B detected at 47% (235 mM) of the imidazole. The proteins were detected by ultra-violet (UV) visible absorption at 280 nm during the 90 min.

**Figure 6:**
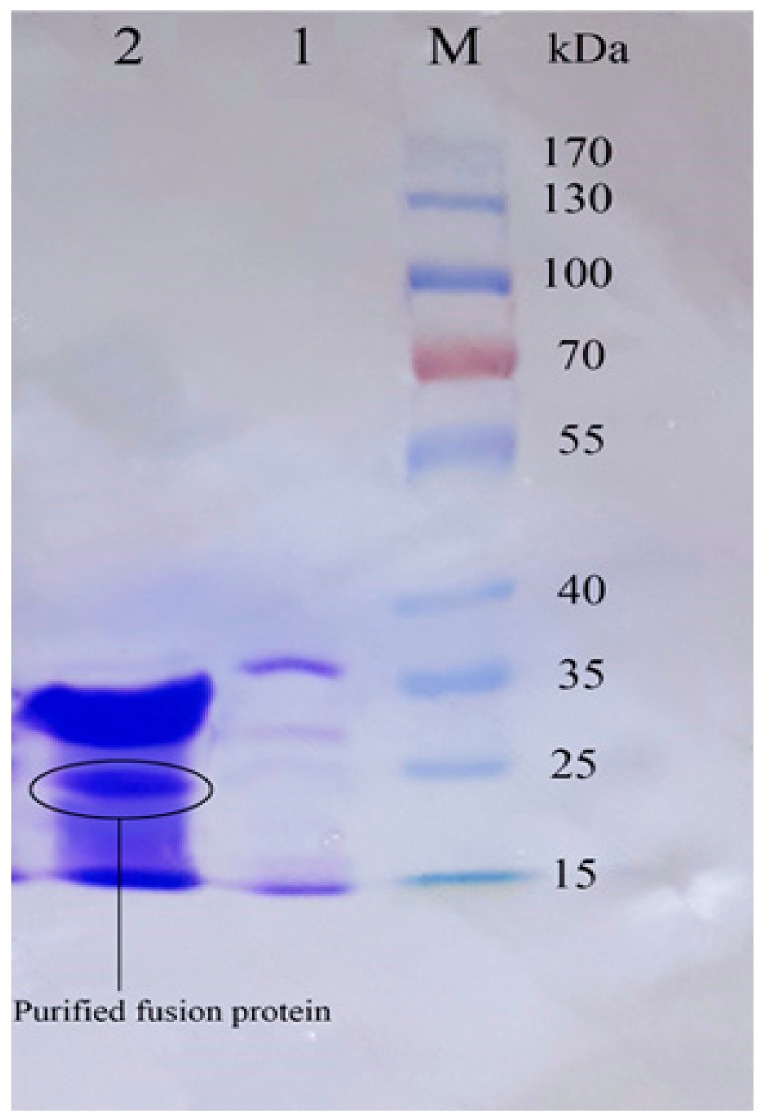
The SDS–PAGE analysis of the eluted protein fractions (according to the diagram in Figure 5) in a gradient concentration of the imidazole. The fractions were concentrated using a PEG. M: pre-stained protein ladder 10–180 kDa; lane 1: the eluted protein fraction (pick A based on Figure 5) at 17% imidazole concentration; lane 2: the eluted protein fraction (pick B based on Figure 5) at 47% imidazole concentration. The Trx-rec- Cath-A was detected in fraction B.

**Figure 7:**
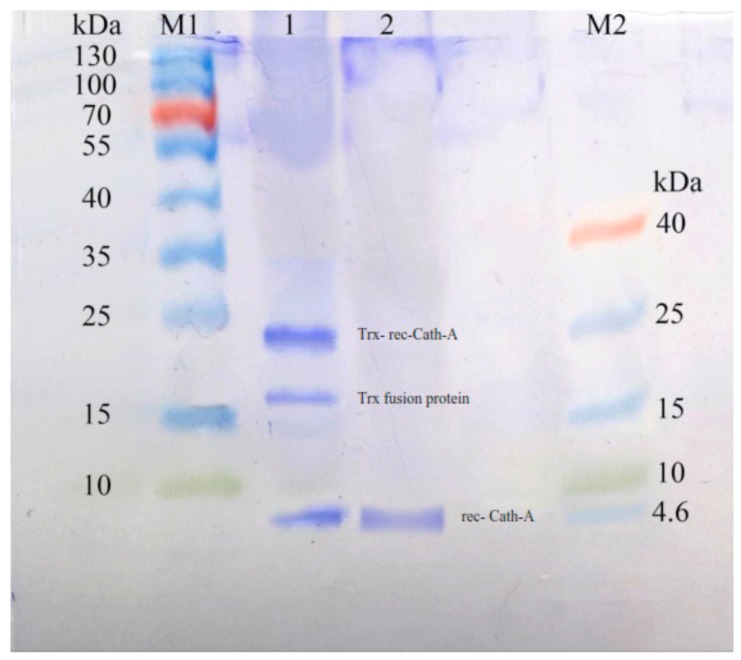
The SDS–PAGE analysis of Trx-rec- Cath-A released by enterokinase cleavage. M1: pre-stained protein ladder 10–180 kDa; lane 1: cleavage reaction mixture including an uncleaved fusion protein (22.8 kDa), a cleaved trxA fusion partner (~16.5 kDa), and Cath-A (4.3 kDa); lane 2: released Cath-A (4.3 kDa) peptide after cleavage by enterokinase, purified by amicon, and after 3 kDa ultrafiltration and dialysis in a phosphate-buffered saline (PBS) 1X buffer, M2: pre-stained low-range protein ladder (1.7–40 kDa).

Using the HisTrap FF affinity column, soluble fusions including Trx–6His–enterokinase–Cath-A were purified in an elution buffer containing 0.5 M imidazole. To detect exactly the fraction that contained rec–Cath-A, a gradient of the imidazole concentration in the elution buffer was used. According to the results, two fractions were eluted at 17% (85 mM) and 47% (235 mM) of the imidazole concentration. The fractions were concentrated using PEG and were analyzed by SDS-PAGE (Figure 5). The desired peptide was concentrated in the second peak (B) which was eluted by the 47% imidazole concentration (Figure 6). The buffer exchange in the presence of the enterokinase buffer was performed for the above-mentioned fraction (fraction B), followed by the Trx–6His–enterokinase–Cath-A cleavage, using enterokinase. To remove the non-specific proteins and the buffer exchange to PBS 1X, amicon ultra-filtration 3 and 30 kDa tubes were used. As a final step in the process and to access the intact Cath-A a dialysis process was done. As the cut-off of the dialysis membranes were 14 and 20 kDa, the intact final peptide (Cath-A) left the dialysis bag and the heavier sequence (Trx–6His–enterokinase) inside. The partly purified peptide was analyzed with SDS-PAGE (Figure 7). The protein concentration in each step was presented in Table 2.

### 3.5. Antimicrobial Activity of the Released Peptide

**Figure 8:**
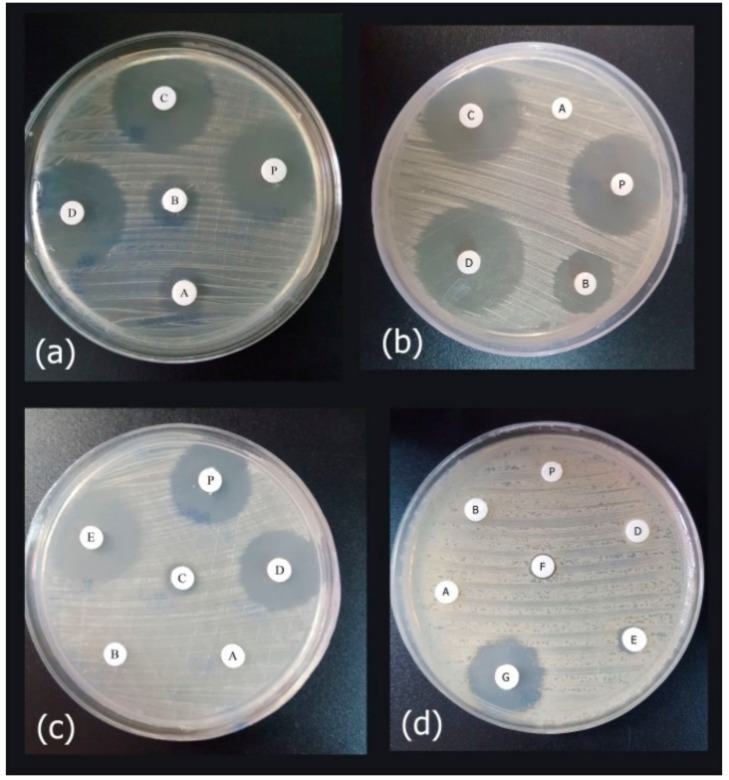
The agar disk diffusion assay of the purified peptide as compared to the synthetic peptide. In each test, approximately 20 μL of purified recombinant peptide (Cath-A) inoculated on a blank disk, P: indicated as a purified peptide in the tests, (**a**) Antimicrobial activity against *E. coli ATCC 25922* (A–D; 2, 4, 8, 16 µg/mL concentrations of the synthetic peptide, respectively), (**b**) Antimicrobial activity against *P. aeruginosa ATCC 27863* (A–D; 2, 4, 8, 16 µg/mL concentrations of the synthetic peptide, respectively), (**c**) Antimicrobial activity against the multidrug resistant (MDR) clinical *A. baumannii no.7* (A–E; 1, 2, 4, 8, 16 µg/mL concentrations of the synthetic peptide, respectively), (**d**) Antimicrobial activity against the MDR clinical *P. aeruginosa no 4* (A–G; 4,8,16,32,64 and 126 µg/mL concentrations of the synthetic peptide, respectively. The higher concentrations (i.e., 256, 500 and 1000 was displayed in Figure S2).

**Figure 9:**
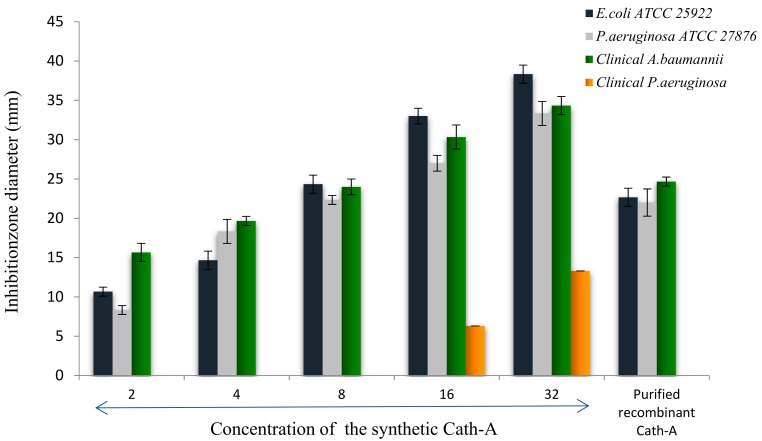
The inhibition zone diameters of the synthetic Cath-A with different concentrations (2–32 µg/mL) against the standard and clinical strains. The zone inhibition of the purified recombinant Cath-A peptide was approximately similar to an 8 µg/mL concentration of the synthetic peptide. Data are reported as mean ± SD of the three independent experiments.

Antimicrobial assays of the released peptide were compared to the different concentrations of the synthetic peptide using the disk diffusion method, against *E. coli* ATCC 25922, *P. aeruginosa* ATCCC 27863, and clinical isolates of *P. aeruginosa* and *A. baumanii* (Figure 8). As the results showed in Figure 8, the expressed purified peptide (17.4 µg/mL) exhibited antimicrobial activity similar to the inhibitory effects of 8 µg/mL of the synthetic peptide (Figure 9).

## 4. Discussion

An imminent need for new antibiotics dictates the necessity for studies aimed at designing clinically useful AMPs. The present study was carried out on such a basis and it provides a 34 amino-acid long AMP sequence named Cath-A. In previous reports, this peptide was proven to be effective in ex vivo studies using different pathogen microorganisms containing standard and wild-type strains, especially the methicillin-resistant *Staphylococcus aureus* (MRSA). Additionally, the cell culture and hemolysis studies proved that the peptide was safe in its effective concentrations [45]. 

Bacterial cells form biofilm matrixes under several environmental conditions including nutritional signs and starvation, attachment to the host tissues or non-living surfaces, exposure to sublethal concentrations of antibiotics, and environmental stresses. The biofilm structures are resistance to the stress, especially the conventional antimicrobial agents and the host defense mechanisms. It is estimated that microbes growing in the biofilm structures are more resistant to antibiotics compared to the planktonic cells. So the treatment of biofilm-related infections is a significant problem in healthcare systems. To clear the biofilm forming bacteria, the various antibiotics classes (e.g., ß-lactams, aminoglycosides or fluoroquinolones) are generally used. Hence, new developed antibacterial agents with alternative strategies are required for the treatment and eradication of established biofilms in clinical settings [57,58,59]. Applying AMPs as an anti-biofilm strategy has been considered, which may represent promising approaches to control biofilms. AMPs have been considered as alternative therapeutic candidates for conventional antibiotics. Their important roles in the modulation of innate host immune defenses, broad-spectrum antimicrobial activity against microorganisms, efficacy on neutralizing lipopolysaccharide endotoxin, rapid mechanism of actions on MDR bacteria, and low incidence in selecting resistance to AMPs are the benefits of these molecules [32,33].

Therefore, in this report, the antimicrobial potential of Cath-A was examined against the *A. baumannii* and *P. aeruginosa* biofilm isolates. Among the opportunistic bacteria, MDR *P. aeruginosa* and *A. baumannii* are considered medically important pathogens. These pathogens are commonly associated with nosocomial and hospital-acquired infections, especially in the ICU and burn sites. [23,60,61]. The strategy of applying AMPs as an anti-biofilm has been considered, which may represent promising approaches to control biofilms [46]. Other researchers have also reported an anti-biofilm efficacy of the AMPs against *A. baumanii* and *P. aeruginosa* [62,63,64]. Most of the medically relevant biofilms are resistant to commercial antimicrobial agents because of their structural and functional properties. In the present study, the isolates exhibited MDR properties in the phenotypic antimicrobial assays, as reported in previous scientific reports [65,66,67]. The anti-biofilm activities of Cath-A were substantial, particularly for the MDR *A. baumanii* isolates. In the case of the *P. aeruginosa* strains, Cath-A had anti-biofilm effects comparable to commercially available antibiotics. However, some isolates among *P. aeroginusa* exhibited a resistance to Cath-A (MIC ≥ 265 µg/mL) which may be related to the bacterial resistance mechanisms such as proteolytic degradation, the modification of cell wall components, or the presence of alginate in the biofilm structure [68]. Additionally, as there are few candidate molecules available against biofilm-related infections, these results suggest the need for more studies in order to evaluate Cath-A as a promising AMP with a potential clinical usefulness. Since the levofloxacin is a CLSI recommended antibiotic, we compared the eradication effects of Cath-A with levofloxacin in biofilm biomass.

Another important issue in this report was the method that was used for the production of Cath-A. The *E. coli* expression system was applied so as to express recombinant Cath-A because of its cost-effectiveness and the over-construction of the desired product. The suitable expression vector (pET 32a) was used as it contains the Trx fusion protein in order to eliminate the toxicity of the peptide and to express the soluble peptide in a correct folding. The existence of the His-tag was an important factor in the purification process using the affinity chromatography from the HisTrap FF column. These properties made the purification easier and increased the product yield. The inserted extra enterokinase sequence at the N-terminus of rec-Cath-A facilitated the cleavage process and ended up being the desired sequence. In the present study, the time of expression after the IPTG induction played a critical role in producing rec- Cath-A. This is in accordance with the reports of other researchers [69,70]. In contrast, increasing the expression time led to a reduction of the rec-Cath-A production after 6 h. The yield of the recombinant peptide was about 0.09 mg from 500 mL of bacterial culture. It was lower than the yield of other expressed AMPs in *E. coli* [71]. This may have resulted in the cytolytic activity of the expressed rec- Cath-A or in the bacterial proteases’ effects on the peptide, which greatly decreased the yields of the products. The rec- Cath-A displayed antimicrobial activities on the MDR isolates. The impurities of the recombinant peptide may cause an increase in the concentration of MIC tests and result in twice as low antimicrobial activity compared to the synthetic peptide. It seems that other expression systems that have been used to express AMPs demonstrate a 100% in vitro activity when compared to their synthetic analogues [72,73].

## 5. Conclusions

In this paper, we have described an expression approach to produce soluble, active, and partially purified recombinant Cath-A in *E. coli* as an expression host. The potential antimicrobial effect of Cath-A against MDR bacteria was demonstrated. Our findings suggest that the Cath-A peptide could be a promising topical candidate to control the MDR biofilm-forming bacteria in the healthcare settings.

## Figures and Tables

**Table 1:** The MICs of the commercial antibiotics and the Cath-A peptide against ATCC standard strains and biofilm formation microorganisms.

Microorganisms	MIC (µg/mL)							
	Ap	PIP	CAZ	CP	T	LEV	IMI	Cath-BF
*P. aeruginosa 1*	>256	>256	256	128	128	64	32	32
*P. aeruginosa 2*	>256	>256	64	256	128	8	8	64
*P. aeruginosa 3*	>256	>256	>256	>256	>256	16	>256	>256
*P. aeruginosa 4*	>256	>256	256	128	128	32	32	128
*P. aeruginosa 5*	>256	>256	16	32	>256	256	32	16
*P. aeruginosa 6*	>256	64	64	64	32	16	8	64
*P. aeruginosa 7*	>256	>256	>256	128	>256	64	256	>256
*P. aeruginosa 8*	>256	>256	>256	64	32	64	32	256
*A. baumannii 1*	>256	>256	256	256	256	16	>256	16
*A. baumannii 2*	>256	>256	128	64	32	16	>256	8
*A. baumannii 3*	>256	>256	>256	128	>256	16	256	8
*A. baumannii 4*	>256	>256	64	32	>256	8	256	8
*A. baumannii 5*	>256	>256	128	64	>256	16	256	8
*A. baumannii 6*	>256	>256	128	128	>256	>256	256	8
*A. baumannii 7*	>256	>256	64	64	64	16	128	16
*A. baumannii 8*	>256	>256	128	32	256	32	256	16
*A. baumannii 9*	>256	>256	>256	128	128	64	>256	32
*A. baumannii 10*	256	>256	128	32	64	32	128	8
*P. aeruginosa* ATCCC 27863	128	4	2	1	32	0.5	4	8
*E. coli* ATCC 25922	2	0.5	0.5	<0.25	0.5	0.25	1	8

MIC: Minimum inhibitory concentration; Ap: Ampicillin; P: Piperacillin; CAZ: Ceftazidime; CP: Cefepime T: Tetracycline; Lev: Levofloxacin, IMI: Imipenem.

**Table 2:** The purification steps and yield of the fusion protein and rec-Cath-A based on a 500 mL culture of *E. coli BL21(DE3)*.

Fraction	Volume (mL)	Concentration (mg/mL)	Total Protein (mg)	Yield (%)
Crude supernatant	60	2.57 ± 0.07	154.6 ± 4.2	100
Ni-NTA Histrap	45	1.42 ± 0.09	64.2 ± 4.2	41.5
Pick-II	20	0.576 ± 0.015	11.52 ± 0.30	7.45
Ultrafilter	10	0.394 ± 0.019	3.94 ± 0.19	3.2
rec-Cath-BF (Dialysis)	5	0.0176 ± 0.008	0.088 ± 0.04	0.34

Data Shown in the table were the average results of three independent experiments. The protein concentration was determined by the standard Bradford protein assay using BSA.

## Supplementary Materials

The following are available online at http://www.mdpi.com/2218-273X/8/4/118/s1, Figure S1: The antibiotic susceptibility tests by disk diffusion method, Figure S2: Antimicrobial activity against MDR clinical *P. aeruginosa* no 4.
