# Supplementary material for: A Recombinant Snake Cathelicidin Derivative Peptide: Antibiofilm Properties and Expression in Escherichia coli
Source: Biomolecules. 2018 Oct 22;8(4):118. doi: 10.3390/biom8040118 (PMC6315654; doi:10.3390/biom8040118)
Supplement: Supplementary file 1 [file biomolecules-08-00118-s001.pdf]

biomolecules

Supplementary Material

A Recombinant Snake Cathelicidin Derivative Peptide: Antibiofilm Properties and Expression in *Escherichia coli*

Mercedeh Tajbakhsh [1], Maziar Mohammad Akhavan [2], Fatemeh Fallah [1] and Abdollah Karimi [1,*]

[1] Pediatric Infections Research Center (PIRC), Research Institute for Children Health, Shahid Beheshti University of Medical Sciences, 1546815514 Tehran, Iran; mercedeh.tajbakhsh@gmail.com or m_tajbakhsh@sbmu.ac.ir (M.T.); fafallah@sbmu.ac.ir (F.F.)

[2] Skin Research Center, Shahid Beheshti University of Medical Sciences, 1989934148 Tehran, Iran; m_akhavan@sbmu.ac.ir

* Correspondence: dr.karimi@sbmu.ac.ir; Tel.: +98-212-222-9641

Received: 8 October 2018; Accepted: 17 October 2018; Published: 22 October 2018

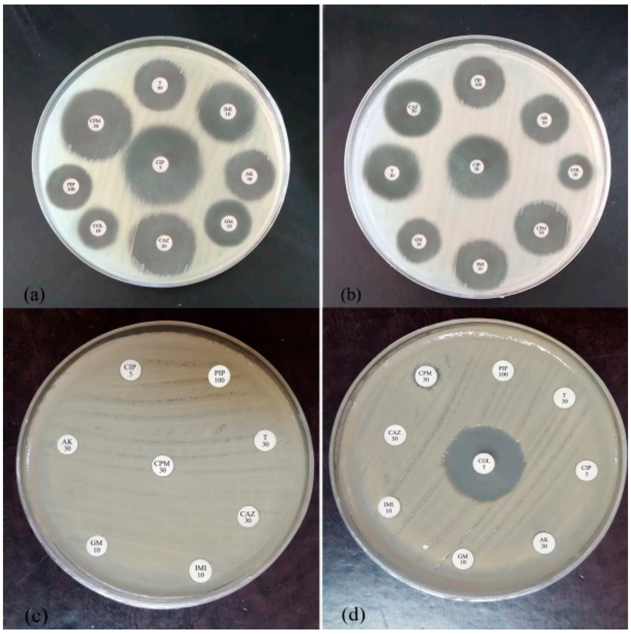

Figure S1. The antibiotic susceptibility tests by disk diffusion method. (a): Antimicrobial activity against *E.coli ATCC 25922*, (b): Antimicrobial activity against *P.aeruginosa ATCC 27863*, (c): Antimicrobial activity against MDR clinical *A.baumannii* no.7, (d): Antimicrobial activity against MDR clinical *P.aeruginosa* no 4. The strains were assessed against the following antibiotic disks: PIP(piperacillin, 100 μg),CAZ(ceftazidime ,30μg), CPM(cefepime, 30μg),IMI (imipenem, 10μg),GM (gentamicin, 10μg), AK(amikacin, 30μg), T(tetracycline, 30μg), CIP(ciprofloxacin, 5μg) and COL (colistin. 5 μg).

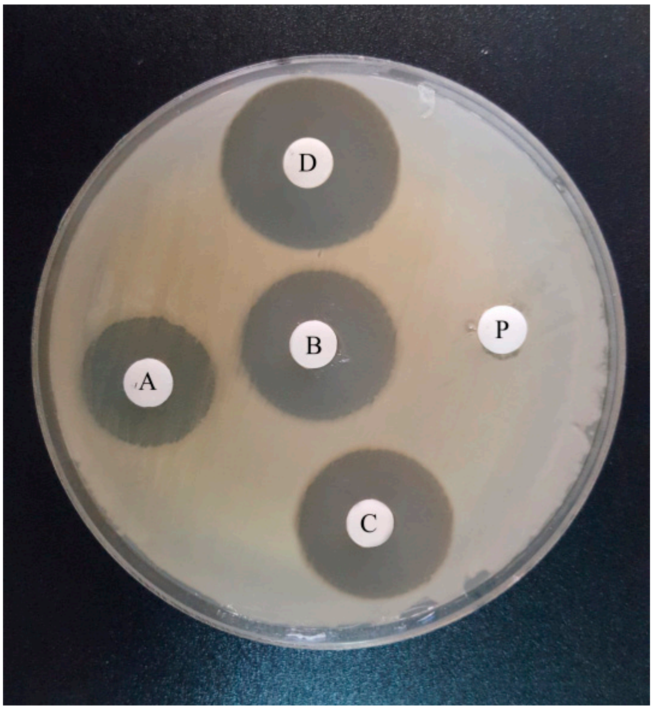

Figure S2. Antimicrobial activity against MDR clinical *P. aeruginosa* no 4 (A-D; 128, 256, 512 and1000 μg/ml concentrations of synthetic peptide respectively, P: purified recombinant Cath-A peptide).

© 2018 by the authors. Submitted for possible open access publication under the terms and conditions of the Creative Commons Attribution (CC BY) license (http://creativecommons.org/licenses/by/4.0/).
